# The relationship between martial arts practice experience and psychological resilience among Chinese college freshmen: the mediating role of self-control and the moderating role of perceived social support

**DOI:** 10.3389/fpsyg.2025.1686000

**Published:** 2025-11-28

**Authors:** Chen Tao, Yue Li

**Affiliations:** 1 College of Physical Education, Yangzhou University, Yangzhou, China; 2 Ministry of Public Sports, Taizhou University, Taizhou, China

**Keywords:** martial arts practice experience, college freshmen, psychological resilience, self-control, perceived social support

## Abstract

**Objective:**

This study aims to examine the moderating effect of perceived social support and the mediating role of self-control in the relationship between psychological resilience and martial arts experience among first-year college students.

**Methods:**

A cluster sampling method was used to select 1,291 university freshmen from Jiangsu Province. Data were collected through a basic information questionnaire, a Martial Arts Practice Experience Questionnaire, a Psychological Resilience Scale, a Self-Control Scale, and a Perceived Social Support Scale.

**Results:**

(1) College freshmen with martial arts practice experience demonstrate greater psychological resilience and self-control than those without martial arts practice experience. (2) Martial arts practice experience was significantly positively correlated with psychological resilience (*r* = 0.447, *p* < 0.01), self-control (*r* = 0.478, *p* < 0.01), and perceived social support (*r* = 0.201, *p* < 0.01). (3) Self-control partially mediated the relationship between martial arts practice experience and psychological resilience (*β* = 0.491, *t* = 19.747, *p* < 0.001), with the mediation effect accounting for 52.57% of the total effect. (4) Perceived social support played a moderating role in the second half of the mediation model (*β* = 0.133, *t* = 6.314, *p* < 0.001), such that the higher the level of perceived social support among college freshmen, the stronger the enhancing effect of self-control on psychological resilience.

**Conclusion:**

The findings revealed a moderated mediating relationship between martial arts practice experience and psychological resilience among college freshmen, with self-control serving as a mediating variable between the two factors. Additionally, perceived social support strengthened the positive effect of self-control on psychological resilience among college freshmen.

## 1 Introduction

### 1.1 Martial arts practice experience and college freshmen psychological resilience

College freshmen are faced with a variety of academic, social, and personally-based problems as they transition into university life. This life phase occurs during late adolescence, a time of continued development, both neurocognitively and psychosocially, which makes students susceptible to stress, anxiety, and depression (Fruehwirth et al., 2024; Reynolds et al., 2011). Psychological resilience (PR)—the ability of an individual to retain adaptive functioning in the context of adversity (Sisto et al., 2019)—represents an important area of investigation and development since it is an important protective factor. Developing the PR of freshman students is of great concern and requires the establishment of appropriate interventions, which are palatable and can be introduced into the university setting (Xia et al., 2017). The design and experience of martial arts practice experience (MAPE) for these students is presented as a strong vehicle for the development of PR. The rationale behind this lies in the fact that the unique and dynamic qualities of this discipline represents both a sport that develops an interindividual finesse, with many training requirements stressing the esthetic qualities of execution but also a combat sport which places strong demands on tactical processes (Tian, 1998). In effect, the training experience becomes one of physical and mental discipline. The practice is based on regularity of the requisite movement trains, obedience to ethical codes (the practice of discipleship and respect, etc.) and personal challenge (Tadesse, 2017). There is a substantial body of evidence demonstrating that martial arts practice can significantly develop PR and self-efficacy in various groups and types of student populations (Moore et al., 2019; Pekel et al., 2025; Filho et al., 2023; Theeboom et al., 2009). In addition to this martial arts practice represents an age-old example of structured exercise which is focused on the development of this very factor self-regulation capacities. The constant requirement for an attention span, as well as an ability to control impulses during combat situations as well as maintaining a certain level of discomfort throughout the duration of drills and routines, brings this type of learning within a direct and contextual setting (SC)—a well-accepted psychological phenomena and inherent part of resilience (Köroğlu et al., 2025). Effectively therefore martial arts represent more than physical options, they represent the opportunity for mental training, which systematically achieves the development of the inner psychological tools (i.e., self- control (SC)). which underlie PR. The freshman population represents a psychotherapeutic population at this very time of development. They represent a group in transition but one where autonomy is continually being broadened, but the abilities to self-control are being developed into their full potential. This makes them specifically predisposed to be susceptible to forms of instruction which can structure and widen these autonomous capacities. The problems of freshmen year—academic pressure, social pressures of integration, identity development etc. represent a naturalistic opportunity for the effectiveness of developed SC and PR which will be relevant and chemically effective in improving mental health and adjustment (LIAO et al., 2019). Therefore, the confluence of martial arts (as a subject of intervention) and freshmen (as a subject group ripe for the opportunities for acceptance of acquisition of these character) presents as a fruitful area for relevant and highly impactful research. If this correlation is to be investigated it will take us away from the vague generalizations regarding the effectiveness of exercise, and lead us toward an understanding of mechanistic developments of the various factors which the careful development of a mind–body discipline can offer in addressing the modern issues of mental ill-health faced in educational institutions.

### 1.2 The mediating role of self-control

Self-control (SC)—the capacity to monitor and regulate one’s thoughts, feelings, and behavior in order to achieve long-term goals (Duckworth et al., 2019)—is hypothesized to be a crucial mediator between martial arts practice and psychological resilience. Martial arts practice necessarily promotes SC due to the training’s demands for focused attention, inhibition of impulses during fighting, and perseverance in learning complex routines (Köroğlu et al., 2025). This has been demonstrated empirically, since training in karate (Guo and Yang, 2022) and judo (Potoczny et al., 2022) has been shown to be a more effective means of promoting SC and other associated executive functions than general aerobic exercise. Of fundamental importance is the fact that SC has a well-deserved reputation as an important foundation of psychological resilience. It allows individuals to retain tranquility under pressure, the opportunity to control emotions, and to persevere in the face of obstacles (Gokalp, 2023; Köroğlu et al., 2025). For entering university freshmen, who must deal with a number of new academic and social settings, having a strong development of SC is critical to guard against academic procrastination and a variety of emotional distress (Powers et al., 2020). Hence, with the systematic development of self-control, martial arts practice provides a more developmental route toward the fostering of psychological resilience.

### 1.3 The moderating role of perceived social support

The shift to university life can drain the psychological resources of first year students. This results in a great need for external support systems. Perceived social support (PSS) is the belief that one has access to emotional, informational and practical help from the social network, which buffers the negative effects of such stress (Lei et al., 2020). The self-determination theory posits that peer/family/institutional support meets the fundamental psychological needs relatedness and competence and, thus, leads to healthier methods of coping (Miles et al., 2022). PSS increases mental health directly as well as indirectly by strengthening psychological resources such as self-control. For example, social support serves to lessen anxiety and depression, in part by increasing self-regulation capabilities (Ti et al., 2025). This implies that PSS can increase the strength of the relationship between SC and psychological resilience. When students feel supported, the self-control gained through martial practice can be more effectively mobilized and sustained in stressful conditions and thus, there can be greater resiliency. Thus, PSS is theorized to act as a moderator, amplifying the benefit of self-control on resilience outcomes. Accordingly, the following research hypotheses are proposed:

*H1:* Self-control mediates the connection of martial arts practice experience with psychological resilience in college freshmen.

**Figure 1:**
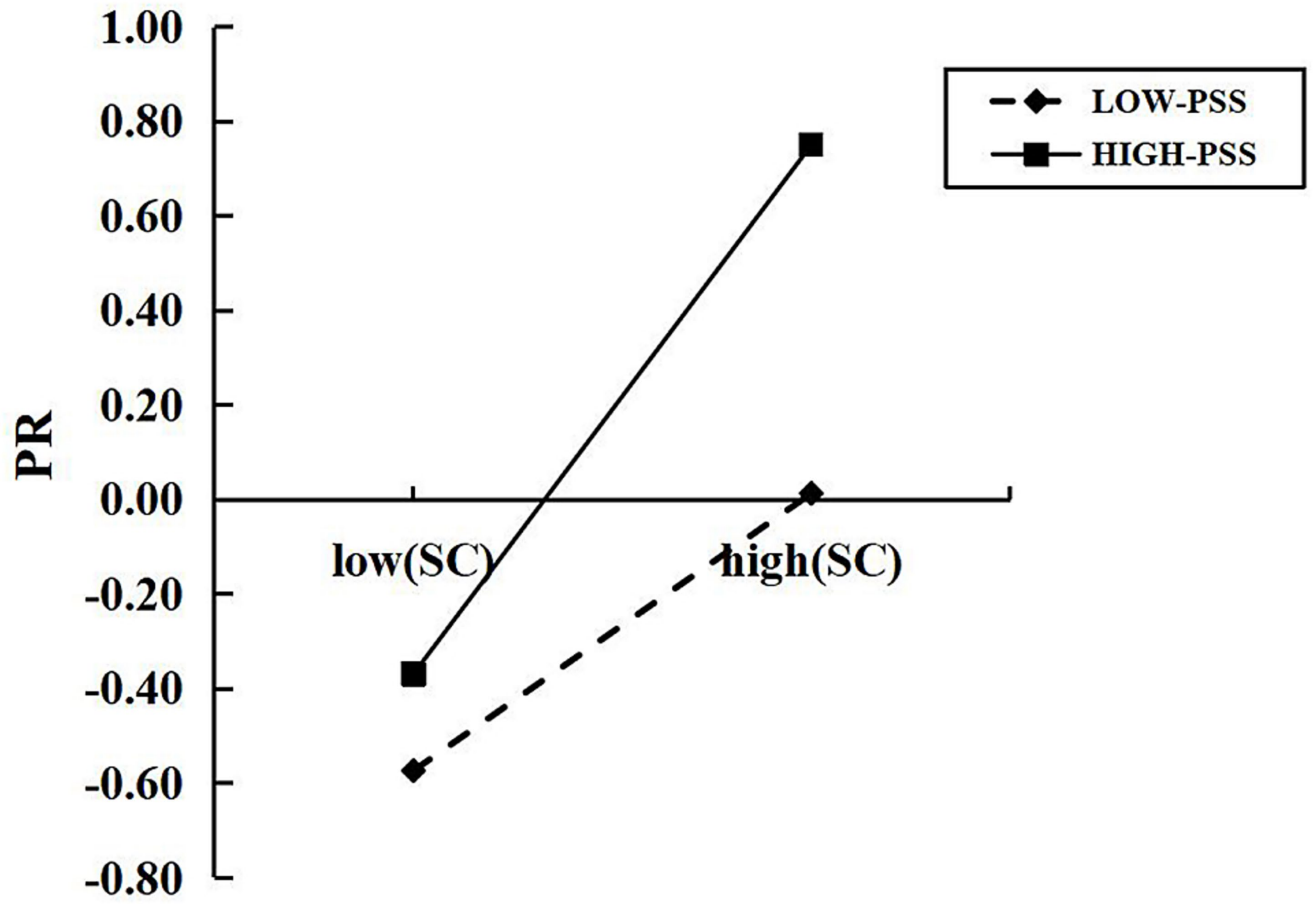
The moderated mediation model.

*H2:* Perceived social support moderates the aforementioned mediating pathway (i.e., the mediating role of self-control between martial arts training experience and psychological resilience in college freshmen), specifically influencing the second half of this pathway (i.e., self-control → psychological resilience). See the model in Figure 1.

## 2 Materials and methods

### 2.1 Participants

This survey targeted first-year students majoring in non-sports disciplines at four representative universities across different regions of Jiangsu Province that recruit students nationwide. Employing a stratified random sampling method, professionally trained instructors administered the survey uniformly within each class. After obtaining participants’ consent, questionnaires were distributed and collected through a combination of online and offline methods. Each questionnaire contained the following elements: a survey collecting basic information about the participants, a martial arts practice experience survey, a Self-Control Scale, a Perceived Social Support Scale, and a Psychological Resilience Scale. A total of 1,553 questionnaires were distributed. After sorting and excluding invalid questionnaires (invalid questionnaire exclusion criteria: irregular completion, incorrect or incomplete answers), 1,291 valid questionnaires were obtained, with a response rate of 83.13%. Among the completed questionnaires, 671 were completed by males and 620 by females, with an average age of 18.3 ± 1.2 years.

### 2.2 Measures

#### 2.2.1 Martial arts practice experience questionnaire

This questionnaire was scored using the self-report method (Guo et al., 2024). The martial arts training experience of the participants in the research had to meet the following three conditions: (1) continuous martial arts practice for 6 months; (2) training frequency of three times per week, with each training session lasting at least 45 min; and (3) the types of martial arts practiced were primarily those offered in school martial arts courses and after-school training programs (excluding martial arts majors), including combat-oriented and performance-oriented such as tai chi, taekwondo, sanda, judo and other martial arts forms. Using a binary categorical variable. Each condition was scored as follows: “yes” received 1 point and “no” received 0 point. A score of 3 points was classified as “With martial arts practice experience,” while those who scored less than 3 points were classified as “Without martial arts practice experience.”

#### 2.2.2 Self-Control Scale

The Self-Control Scale (SCS) was carefully translated and optimized by Shuhua and Yongyu (2008). The scale comprises 19 items dealing with five different angles of self-regulation, impulse control, adherence to healthy habits, resistance to temptation, work concentration, and personal recreation. The answers are scored on a five-point Likert type scale from 1 (“entirely disagree”) to 5 (“entirely agree”) to quantify and asses a quality of SC of the individual. The higher the scoring the better the SC is. In the study, the Cronbach’s *α* coefficient for the total scale was 0.929.

#### 2.2.3 Perceived Social Support Scale

The Perceived Social Support Scale (PSSS), which Zimet et al. (1988) developed was used to evaluate college students’ social support and social life. It has 12 items and 3 dimensions. Specifically, it has 4 items on family support, 4 items on friend support, and 4 items on other support. There are no reverse scored items. The replies are on a 7-point Likert scale: 1 (strongly disagree), to 7 (agree), with higher scores representing a stronger and higher quality social connection of college students to society. In this study, for the total scale, the value of Cronbach’s a was 0.876.

#### 2.2.4 Psychological Resilience Scale

The Psychological Resilience Scale, revised by Xiaonan and Jianxin (2007), consists of three dimensions and 25 items. There are eight items on self-reliance, four items on optimism, and 13 items on resilience. Responses are scored on a five-point Likert scale, from 1 (“strongly disagree”) to 5 (“agree”). Higher scores indicate higher PR. In the current study, the Cronbach’s *α* coefficient for the total scale was 0.941.

### 2.3 Statistical analysis

SPSS 27.0 was used to conduct reliability and validity tests, compile descriptive statistics, and run a Pearson correlation analysis to explore the relationships among martial arts practice experience, college students’ SC, PSS, and PR variables. Model 4 in the PROCESS 4.1 macro program and the Bootstrap method (Hayes, 2013) were used to test the mediating effect of SC between martial arts practice experience and college students’ PR. The moderating effect of PSS was tested via Model 14 in the PROCESS 4.1 macro program and the Bootstrap method. The significance level was set at *α* = 0.05. Based on the results of the skewness tests and kurtosis in this paper, it is found that the absolute value of skewness is 0.068, and the absolute value of kurtosis is 0.136, and they are both less than 2, so it can be inferred that the distribution of data conforms to the requirement of normality and can carry out the subsequent research.

## 3 Results

### 3.1 Common method bias test

This study employed self-report methods for data collection, necessitating testing for common method bias. During testing, common method bias was procedurally controlled through anonymous measurement and the use of partially reverse-scored items. All variables involved in this study—martial arts practice experience, psychological resilience among college freshmen, self-control, and perceived social support—underwent KMO tests and Bartlett’s sphericity tests. Results showed KMO = 0.965 and Bartlett’s value of 45,581.187, with *p* < 0.01, indicating the data is suitable for factor analysis. Factor analysis was conducted using Harman’s one-factor test. The findings yielded six exploratory factors with eigenvalues above the value of 1, with the maximum variance of a factor being 29.681%, which is below the 40% level. Furthermore, there were no cases of only one factor being extracted, indicating that there were no severe common method bias problems, and that further analysis could be conducted.

### 3.2 With/without martial arts practice experience differences in each main variables

With/Without Martial Arts Practice Experience differences in each main variables were investigated using the Independent samples *t*-test. As presented in Table 1, With Martial Arts Practice Experience college freshman’s self-control (67.15 ± 13.551) and psychological resilience (86.61 ± 17.716) showed higher than Without Martial Arts Practice Experience student’s (52.95 ± 13.931, 69.84 ± 17.804). At the same time, *p* < 0.05 indicates significant statistical significance.

**Table 1:** With/without martial arts practice experience differences in each main variables (M ± SD).

	SC	PR
With martial arts practice experience	67.15 ± 13.551	86.61 ± 17.716
Without martial arts practice experience	52.95 ± 13.931	69.84 ± 17.804
*t*	−18.318	−16.768
*p*	<0.001	<0.001

SC, self-control; PR, psychological resilience; M, mean; SD, standard deviation.

### 3.3 Descriptive statistics and correlation analysis

Table 2 presents the means, standard deviations, and correlation results for the variables of martial arts practice experience and college students’ SC, PSS, and PR. The findings revealed statistically significant positive relationships between all combinations of the four variables. Then, for the sake of studying the correlation among the variables, the Pearson correlation analysis is used. Analysis revealed highly significant positive correlations between MAPE and PR (*r =* 0.447, *p* < 0.01), MAPE and SC (*r* = 0.478, *p* < 0.01), and MAPE and PPS (*r* = 0.201, *p* < 0.01); PR and SC (*r* = 0.592, *p* < 0.01), PR and PPS (*r* = 0.408, *p* < 0.01), and SC and PPS (*r* = 0.336, *p* < 0.01) also exhibited highly significant positive correlations.

**Table 2:** Descriptive statistics and correlation analysis.

Variables	M ± SD	1	2	3	4
1. MAPE	2.14 ± 1.115	1			
2. SC	61.12 ± 15.402	0.478^**^	1		
3. PSS	36.13 ± 9.135	0.201^**^	0.336^**^	1	
4. PR	79.49 ± 19.586	0.447^**^	0.592^**^	0.408^**^	1

MAPE, martial arts practice experience; SC, self-control; PSS, perceived social support; PR, psychological resilience; M, mean; SD, standard deviation. All variables in the model were standardized; * *p* < 0.05; ** *p* < 0.01; *** *p* < 0.001.

### 3.4 Mediation analysis

To test the first hypothesis of the study, Model 4 (MAPE→SC → PR) based on the mediating effect was analyzed. The mediating role of SC was examined using martial arts practice experience as the independent variable and PR as the dependent variable. The results (see Table 3) showed that martial arts practice experience positively predicted PR (*β* = 0.447, *p* < 0.001) (95% CI: 0.398–0.496). After introducing SC as the mediating variable, the positive predictive effect of martial arts practice experience on PR remained significant (*β* = 0.212, *p* < 0.001) (95% CI: 0.163–0.260), while SC significantly predicted PR (*β* = 0.491, *p* < 0.001) (95% CI: 0.442–0.540) and martial arts practice experience significantly predicted SC (*β* = 0.479, *p* < 0.001) (95% CI: 0.431–0.527).

**Table 3:** Regression analysis of the mediating effect of self-control.

OV	PV	*R*	*R* ^2^	*F* (df)	*β*	*t*	95% CI
PR	MAPE	0.447	0.199	321.104^***^	0.447	17.919^***^	0.398–0.496
SC	MAPE	0.479	0.229	382.716^***^	0.479	19.563^***^	0.431–0.527
PR	MAPE	0.621	0.386	403.967^***^	0.212	8.504^***^	0.163–0.260
	SC				0.491	19.747^***^	0.442–0.540

OV, outcome variable; PV, predictor variable; MAPE, martial arts practice experience; PR, psychological resilience; SC, self-control.

The Bootstrap method test for the mediating effect is shown in Table 4. SC played a partial mediating role (95% CI: 0.202–0.269), with the mediating effect accounting for 52.57% of the total effect. These results support the first hypothesis (H1).

**Table 4:** Mediation effect of SC.

PR	Effect	BootSE	BootLLCI	BootULCI
Total effect of PR	0.447	0.025	0.398	0.496
Direct effect of PR	0.212	0.025	0.163	0.260
Mediation effect of SC	0.235	0.017	0.202	0.269

### 3.5 Moderated mediation analysis

To examine the moderating role of PSS in the influence of martial arts practice experience on SC and PR, a moderated mediation analysis was conducted using Model 14 of the PROCESS 4.1 macro. The results (see Table 5) showed that the interaction term between SC and PSS significantly predicted PR (*β* = 0.133, *t* = 6.314, *p* < 0.001), indicating that PSS moderated the second half of the mediation process.

**Table 5:** Moderated mediation analysis results.

OV	PV	*R*	*R* ^2^	*F*	*β*	*t*	95% CI
SC	MAPE	0.479	0.229	382.716^***^	0.479	19.563^***^	0.431–0.527
PR	MAPE	0.670	0.448	261.105^***^	0.203	8.600^***^	0.157–0.250
	SC				0.426	17.347^***^	0.378–0.475
	PSS				0.236	10.673^***^	0.192–0.279
	SC × PSS		0.017	39.869^***^	0.133	6.314^***^	0.092–0.175

OV, outcome variable; PV, predictor variable; MAPE, martial arts practice experience; SC, self-control; PR, psychological resilience; PSS, perceived social support; * *p* < 0.05; ** *p* < 0.01; *** *p* < 0.001.

We examined, via the Bootstrap approach, the indirect effect of the SC on the relationship between the martial arts practice experience (MAPE) and PR, taking into account low, moderate, and high levels of the PSS in analyzing the results. The level of PSS was classified as low, moderate, and high with effect sizes of 0.140 (95% CI: 0.106–0.176), 0.204 (95% CI: 0.172–0.237) and 0.215 (95% CI: 0.226–0.310), respectively. The results are presented in Table 6 and Figure 1.

**Table 6:** Conditional indirect effects by perceived social support level.

ML	IE	BootSE	Bootstrap 95%CI	
			BootLLCI	BootULCI
Low (M−SD)	0.140	0.018	0.106	0.176
Medium (M)	0.204	0.017	0.172	0.237
High (M + SD)	0.268	0.215	0.226	0.310

ML, moderator level; IE, indirect effect.

**Figure 2:**
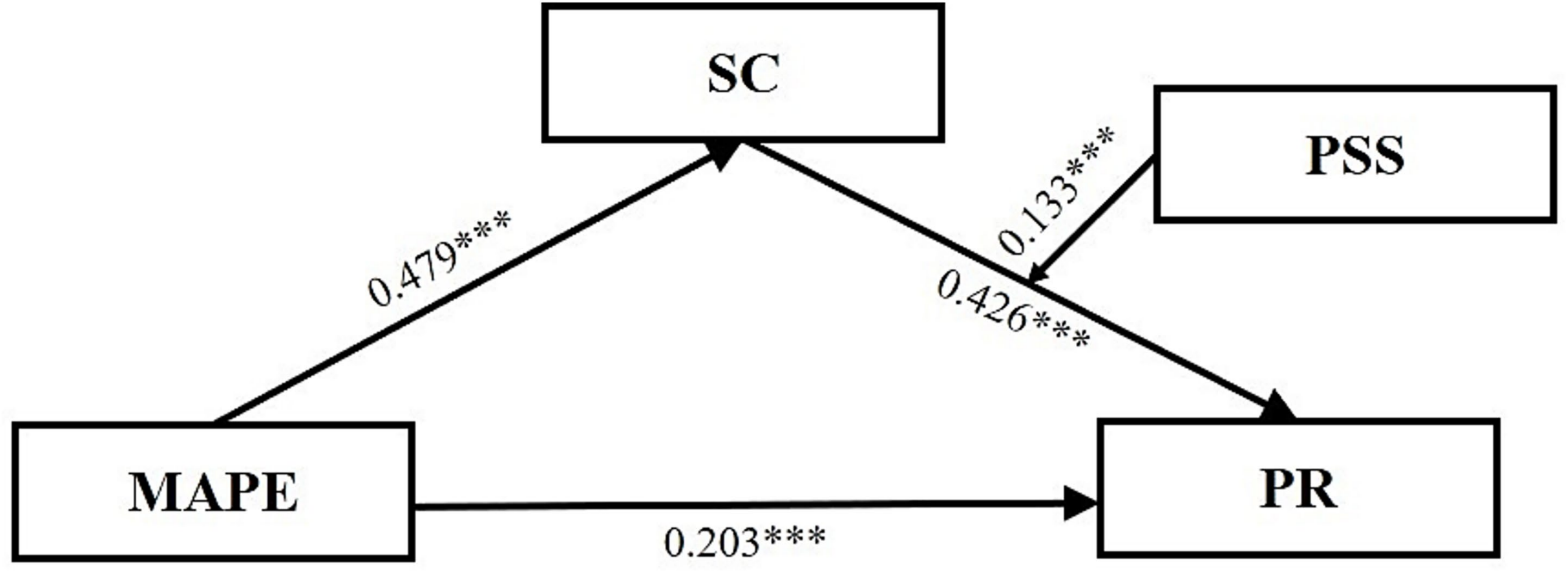
Moderating effect of PSS on the relationship between SC and PR.

The moderating role of PSS was then analyzed by means of further simple slope tests. In the low-PSS group, the PR of college students increased with increasing SC. In the high-PSS group again the PR also increased with increasing SC, but more so than in the former group. This establishes that with greater PSS the facilitative effect of SC on PR increases in strength (see Figure 2). These results are compatible with Hypothesis (H2).

## 4 Discussion

The current study explored the complex pathways through martial arts experience predicts psychological resilience among Chinese college freshmen. The results demonstrate that martial arts experience predicted higher levels of psychological resilience both directly and indirectly via the mediation of self-control. In addition, perceived social support was found to be a significant moderator of the effect of self-control on resilience in such a manner as to strengthen the effect of self-control on resilience. This moderating mediation model further represents a more complete understanding of the resources available to people and the interactions of both social and personal resources resulting in specific resilience outcomes.

### 4.1 The direct and mediated pathways from martial arts practice to resilience

In line with previous studies (Moore et al., 2019), our findings show a significant positive association between martial arts practice experience and psychological resources in first year college students. The mediation analysis shows that the mechanism underlying this relationship is important. Martial arts practice, with its repeated movements, restraint from impulsivity in fighting and adherence to moral (ethical) values such as self-discipline, provides an organized training ground for the self-control (Köroğlu et al., 2025; Tadesse, 2017). This improved self-control ability allows individuals to regulate their emotions, maintain efficiency under pressure and continue working toward long-term goals. Therefore, it is one of the fundamental variables involved in psychological resources (Cai et al., 2022; Köroğlu et al., 2025). This mediational process helps show that the benefits of martial arts are beyond physical fitness and therefore help systemically build much-needed psychological resources.

### 4.2 The significant direct effect and alternative pathways

Self-control was found to partially mediate the effects of martial arts practice on resilience since a significant direct effect of martial arts practice on resilience remained (*β* = 0.212, *p* < 0.001). This indicates that self-control cannot fully illuminate the underlying mechanisms related to resilience. The direct path indicates that other inherent qualities of martial arts training promote resilience. First of all, from a physical literacy perspective, the well developed physicality, balance and coordination, and virtuous capacity to deal with stress that emerges from martial arts training provides a base level of mastery and self-efficacy that can manifest in a strong sense of self-confidence and resilience in dealing with psychological challenges (Li et al., 2024; Whitehead, 2001). Secondly, the philosophical aspects of many martial arts such that “the mind and body work in harmony” and “victory and defeat become transcended” can invoke adaptive patterns of thought as well as balanced mental functioning. The internalization of this philosophy through training may well facilitate both constructive cognitive reappraisal during the situational assessment and execution of positive behavior during psychological adversity, reinforcing psychological resilience (Oh et al., 2025). Future research should probe these mediators, for example physical self-efficacy and stress-related physiological reactivity, to elucidate the more comprehensive natures of the varied routes through which martial arts engenders psychological resilience.

### 4.3 The moderating role of perceived social support

The moderation effect of perceived social support (*β* = 0.133, *p* < 0.001) is statistically significant but represents a small effect size by conventional standards. Several reasons, however, offer evidence of its theoretical and practical relevance. First, in social-psychological systems of a complex nature and involving variables serving as moderators, small interaction effects are often found to be both common and significant in that they are indicative of conditional statements as to the conditions under which main effects appear. Second, inspection of the conditional indirect effects (Table 6) indicates that the disparity in effect size of the indirect effects arising in a low social support condition as compared with a high condition (0.140 versus 0.268) represents an increase of 91% in the strength of the mediation pathway which is substantive and significant from an intervention standpoint. From a theoretical standpoint, the identification of perceived social support as a significant moderator irrespective of the magnitude of effect size offers important theoretical insight for resilience theory inasmuch as it indicates that these psychological benefits derived from martial arts training are not equally distributed among all subjects but are markedly influenced by social contexts. This finding is congruent with the tenets of ecological systems theory and has important implications with respect to the design of interventions that are contextually sensitive.

Our results also show that the positive effects that self-control has on resilience depend on how much perceived social support there is in offered by the person. According to the Conservation of Resources theory (Hobfoll, 2001), people try to get and hold on to what they perceive to be important resources. Self-control can be seen as a very important internal resource that can be depleted with the effects of stress (Chen et al., 2024). In this case perceived social support from family, friends and others can be seen as a very important external resource. For freshmen entering university who have to go through this stressful period, high levels of social support may help offer them the emotional support and practical help necessary to ensure that the self-control resources they have will not be depleted by the academic and social stress they are undergoing (Xu et al., 2023). Thus, it is when self-control resources are retained and replenished by a supportive social environment, that in these people the self-control that they do possess can be effectively used to cope with the stresses that they come up against, resulting in a much more enhanced positive effect that the self-control has on their psychological resiliency. This moderating effect highlights the fact that the psychological benefits that personal mastery type activities such as martial arts have to offer are very closely housed and bolstered in the social environment of the individual.

## 5 Implications and future directions

### 5.1 Theoretical implications

The present results add to the existing knowledge base in sports psychology and positive youth development through an understanding of the mechanism of a given physical activity—martial arts—with regard to psychological resilience. First, this study goes beyond the simple establishment of a correlation by proposing and establishing a moderated mediation model. In the present study, it is shown that self-control is not merely an outcome of martial arts training but is an important psychological mechanism mediating the influence of physical training on resilience. This finding provides more depth to theories of psychological resilience by providing a key self-regulatory pathway by which structured physical disciplines encourage adaptive coping capacities. Secondly, the moderating influence of perceived social support emphasizes the interactive quality of internal and external resources in fostering resilience. Our results indicate that the benefits of developed self-control are not experienced in a vacuum, as it were, but are magnified by the provision of social support. This integrates ecological systems theory and resource principles into the sports psychology literature because it shows how the psychological benefits of individual-centered activities (e.g., martial arts) are situated within the social ecosystem of the individual and are enhanced by it (Bronfenbrenner, 2000; Wilson et al., 2013). Collectively, these findings present a more finely-grained, process-oriented theoretical framework for understanding how mind–body practices like martial arts contribute to adolescent development, emphasizing the synergy between personal mastery and social resources.

### 5.2 Practical implications

The results of this study offer actionable insights for university administrators, mental health practitioners, and physical education policymakers.

#### 5.2.1 For university mental health promotion

In light of the recognized connection between the practice of martial arts, self-control and resilience, institutions of higher education would do well to consider the formal incorporation of martial arts into mental health and freshmen orientation programs. One means of doing this would be to offer martial arts as a credited physical education experience or by means of a structured extracurricular activity. Martial arts could and should be encouraged as a preventive, participatory and non-stigmatizing means of increasing students’ psychological resources at a particularly significant period of development.

#### 5.2.2 For martial arts instruction and program design

We need to make coaches and instructors aware of the powerful psychological benefits of what they are teaching. The didactics of the program can be carefully formulated in such a way that self-control is acquired and trained in a deliberate manner. Thus, the different elements of the training (keeping attention in kata, controlling impulses in sparring) can be proficiently connected to desirable states in life in general. It is of equal importance that a proper sports community is established, as our study proves that a high-quality social environment enhances the strengthening effects of the self-control qualities which are being cultivated in the training.

#### 5.2.3 For home-school collaboration

It is important for universities to communicate these findings to parents to encourage them to allow their children to participate in structured activities. In addition, a system of peer support networks should be established among the freshmen classes to create the level of perceived social support that would increase the amount of personal growth realized through individual effort and discipline.

### 5.3 Limitations and future research

Despite some valuable contributions, this study is subject to several limitations. First of all, the operationalization of martial arts practice experience’ was treated as a yes/no variable, which while methodologically straightforward, has a lack of granularity. Future research should use more sensitive, continuous metrics for such experiences like total cumulative practice hours, years of experience or levels of skill proficiency, as a better means of assessing the degree and/or intensity of experiences involved in martial arts practice. Second, the research does not deal with the study of different martial arts styles, and a continued systematic and comparative study of the effects on the psychological variables involved would be useful, differentiating for example the effects on psychological variables of internal (e.g., Tai Chi practice) as distinct from external styles (e.g., Taekwondo practice), traditional martial arts and modern competitive martial arts, styles emphasizing the meditative and self-cultivation aspects as opposed to practical combat skills. Third, future research should seek to broaden the dimensionality of the measurements involved, moving from a simple practice presence measurement process, to a more multidimensional way of measuring practices, by building up a new measurement profile, including variables such as practice philosophy alignment, style of instruction, intensity of practice experience, social atmosphere, through which it might be possible at a more complex level to tease out the psychological mechanisms are at work. In addition, the results achieved from samples deriving from a singular cultural background indeed require more rigorous cross-validation in terms of their generalizability across cultures. Thus, future studies are recommended to replicate the findings of this study with samples deriving from different experiential cultural backgrounds, so adding theoretically involving richness to understanding how cross-cultural factors operate in the minds and behaviors of martial artists in particular and mind–body trainers in general. Such research could be achieved by sampling participants who were training systematically in different forms of the martial arts in a variety of countries, as a means of testing the cultural boundaries of the theoretical model as it has been set out in this study.

## 6 Conclusion

The findings showed that martial arts practice experience positively predicted the level of PR among university freshmen, while SC mediated the connection of martial arts practice experience and psychological resilience. PSS moderated the second half of the mediation path, strengthening the effect of SC on PR. This indicates that martial arts practice experience can enhance PR among university freshmen through SC while also influencing PR through the moderating effect of PSS on SC.

## Data Availability

The raw data supporting the conclusions of this article will be made available by the authors, without undue reservation.

## References

[ref1] Bronfenbrenner U. (2000). “Ecological systems theory” in ed. A. E. Kazdin Encyclopedia of psychology, vol. 3. doi: 10.1037/10518-046

[ref2] Cai X. Gui S. Tang Y. Zhang S. Xu M. (2022). The impact of parenting styles on resilience of adolescent: the chain mediating effect of self-control and regulatory focus. Psychol. Dev. Educ. 38, 505–512. doi: 10.16187/j.cnki.issn1001-4918.2022.04.06

[ref3] Chen F. Wang J. Gao H. Zeng Y. Li Z. Zou H. (2024). The relationship between ostracism and negative risk-taking behavior: the role of Ego depletion and physical exercise. Front. Psychol. 15:1332351. doi: 10.3389/fpsyg.2024.1332351, PMID: 38328375 PMC10847524

[ref4] Duckworth A. L. Taxer J. L. Eskreis-Winkler L. Galla B. M. Gross J. (2019). Self-control and academic achievement. Annu. Rev. Psychol. 70, 373–399. doi: 10.1146/annurev-psych-010418-103230, PMID: 30609915

[ref5] Filho J. A. Z. Gama D. R. N. D. Castro J. B. P. D. Vale R. G. D. S. (2023). Analysis of the associations between self-esteem and resilience of Krav Maga practitioners. Ido Mov. Cult. 23, 29–35. doi: 10.14589/ido.23.4.4

[ref6] Fruehwirth J. C. Huang L. Tompson C. E. Perreira K. M. (2024). Mental health symptoms among us college students before, early, and late into the Covid-19 pandemic: a longitudinal analysis. J. Adolesc. Health 76, 246–253. doi: 10.1016/j.jadohealth.2024.09.026 39520464 PMC11738670

[ref7] Gokalp Z. S. (2023). Examining the association between self-control and mental health among adolescents: the mediating role of resilience. School Psychol. Int. 44, 649–667. doi: 10.1177/01430343231182392

[ref8] Guo Y. Liu Y. Han J. (2024). Martial arts practice experience and bullying risk in junior high school students: the chain mediating role of psychological resilience and self-efficacy. Sports Sci. 45, 85–93. doi: 10.13598/j.issn1004-4590.2024.05.009

[ref9] Guo T. Yang J. (2022). The influence of tai chi intervention on inhibitory function of African college students. Int. J. Phys. Act. Health 1:9. doi: 10.18122/ijpah.1.2.9.boisestate

[ref10] Hayes A. F. (2013). Introduction to mediation, moderation, and conditional process analysis. J.Educ.Meas. 51, 335–337. doi: 0.1111/jedm.12050

[ref11] Hobfoll S. E. (2001). The influence of culture, community, and the nested-self in the stress process: advancing conservation of resources theory. Appl. Psychol. 50, 337–421. doi: 10.1111/1464-0597.00062

[ref12] Köroğlu M. Yılmaz C. Tan Ç. Çelikel B. E. Budak C. Kavuran K. . (2025). Judo exercises increase emotional expression, self-control, and psychological resilience. Front. Psychol. 16:1632095. doi: 10.3389/fpsyg.2025.1632095, PMID: 40735181 PMC12305810

[ref13] Lei J. Ashwin C. Brosnan M. Russell A. (2020). Differences in anxieties and social networks in a group-matched sample of autistic and typically developing students transitioning to university. Autism 24, 1138–1151. doi: 10.1177/1362361319894830, PMID: 31852210 PMC7433695

[ref14] Li N. Wang D. Zhao X. Li Z. Zhang L. (2024). The association between physical exercise behavior and psychological resilience of teenagers: an examination of the chain mediating effect. Sci. Rep. 14:9372. doi: 10.1038/s41598-024-60038-1, PMID: 38654069 PMC11039466

[ref15] Liao J. Bai R. Luo C. (2019). The impact of positive psychological capital on anxiety of freshmen. Chin. J. Health Psychol. 27, 1085–1088. doi: 10.13342/j.cnki.cjhp.2019.07.029

[ref16] Miles S. Ciannella C. Ryan R. M. Deci E. L. (2022). Self-determination theory. Contemp. Educ. Psychol. 61, 1–7. doi: 10.1016/j.cedpsych.2020.101860

[ref17] Moore B. Dudley D. Woodcock S. (2019). The effects of martial arts participation on mental and psychosocial health outcomes: a randomised controlled trial of a secondary school-based mental health promotion program. BMC Psychol. 7:60. doi: 10.1186/s40359-019-0329-5, PMID: 31511087 PMC6737629

[ref18] Oh Y. T. Ryu M. A. Uhm J. P. (2025). The role of intramural combat martial arts in enhancing well-being among international students: a combined theoretical approach. Front. Psych. 16:1582731. doi: 10.3389/fpsyt.2025.1582731, PMID: 40291518 PMC12023476

[ref19] Pekel A. Turan M. B. Eraslan M. Iqbal M. Pepe O. Yoka K. . (2025). Building resilience through self-defense: the role of martial arts in enhancing psychological strength among women. Front. Psychol. 16:1592326. doi: 10.3389/fpsyg.2025.1592326, PMID: 40584067 PMC12202409

[ref20] Potoczny W. Herzog-Krzywoszanska R. Krzywoszanski L. (2022). Self-control and emotion regulation mediate the impact of karate training on satisfaction with life. Front. Behav. Neurosci. 15:802564. doi: 10.3389/fnbeh.2021.802564, PMID: 35095440 PMC8792757

[ref21] Powers J. P. Moshontz H. Hoyle R. H. (2020). Self-control and affect regulation styles predict anxiety longitudinally in university students. Collabra Psychol. 6:11. doi: 10.1525/collabra.280

[ref22] Reynolds E. K. Macpherson L. Tull M. T. Baruch D. E. Lejuez C. W. (2011). Integration of the brief Behavioral activation treatment for depression (Batd) into a college orientation program: depression and alcohol outcomes. J. Couns. Psychol. 58, 555–564. doi: 10.1037/a0024634, PMID: 21787070 PMC4104126

[ref9001] Shuhua T. Yongyu G. (2008). Revision of Self-Control Scale for Chinese College Students. Chin J Clin Psychol, 468–470. doi: 10.16128/j.cnki.1005-3611.2008.05.022

[ref23] Sisto A. Vicinanza F. Campanozzi L. Ricci G. Tartaglini D. Tambone V. (2019). Towards a transversal definition of psychological resilience: a literature review. Medicina 55:745. doi: 10.3390/medicina55110745, PMID: 31744109 PMC6915594

[ref24] Tadesse M. E. (2017). Martial arts and adolescents: using theories to explain the positive effects of Asian martial arts on the well-being of adolescents. Ido Mov. Cult. J. Martial Arts Anthropol. 17, 9–23. doi: 10.14589/ido.17.2.2

[ref25] Theeboom M. Knop P. D. Vertonghen J. (2009). Experiences of children in martial arts. Eur. J. Sport Soc. 6, 19–35. doi: 10.1080/16138171.2009.11687825

[ref26] Ti Y. Yi C. Wei J. Shi G. (2025). Longitudinal associations between academic and social adjustment among Chinese college freshmen: the mediating role of depressive symptoms. Emerg. Adulthood 13, 663–674. doi: 10.1177/21676968251319361

[ref27] Tian M. (1998). Xiangqun training theory. Beijing: People’s Sports Publishing House.

[ref28] Whitehead M. (2001). The concept of physical literacy. Eur. J. Phys. Educ. 6, 127–138. doi: 10.1080/1740898010060205

[ref29] Wilson D. S. Ostrom E. Cox M. E. (2013). Generalizing the core design principles for the efficacy of groups. J. Econ. Behav. Organ. 90, S21–S32. doi: 10.1016/j.jebo.2012.12.010

[ref30] Xia L. Gu R. Zhang D. Luo Y. (2017). Anxious individuals are impulsive decision-makers in the delay discounting task: an Erp study. Front. Behav. Neurosci. 11:5. doi: 10.3389/fnbeh.2017.00005, PMID: 28174528 PMC5258725

[ref9003] Xiaonan Y. Jianxin Z. (2007). Comparative Application of the Self-Resilience Scale and the Connor-Davidson Resilience Scale. J. Psychol. Sci., 1169–1171 doi: 10.16719/j.cnki.1671-6981.2007.05.035

[ref31] Xu Y. Ye Y. Zhen R. Zhou X. (2023). School bullying victimization and post-traumatic stress symptoms in adolescents: the mediating roles of feelings of insecurity and self-disclosure. BMC Psychol. 11, 1–7. doi: 10.1186/s40359-023-01065-x 36721248 PMC9890857

[ref9002] Zimet G. Dahlem N. Zimet S. Farley G. (1988). The Multidimensional Scale of Perceived Social Support. J. Pers. Assess., 52. doi: 10.1207/s15327752jpa5201_2 2280326

